# A maverick: Environmentally relevant concentrations of nonylphenol attenuate the plasmid-mediated conjugative transfer of antibiotic resistance genes

**DOI:** 10.1016/j.wroa.2024.100241

**Published:** 2024-07-26

**Authors:** Si-Zhou Liang, Ya-Jun Chang, Philip Semaha, Li-Zhu Liu, Yan Gao, Zhi Wang, Wei-Guo Zhang

**Affiliations:** a China Ministry of Agriculture Key Laboratory at Yangtze River Plain for Agricultural Environment, Institute of Agricultural Resources and Environment, Jiangsu Academy of Agricultural Sciences, Nanjing 210014, China; b School of the Environment and Safety Engineering, Jiangsu University, Zhenjiang 212013, China; c Jiangsu Key Laboratory for the Research and Utilization of Plant Resources, Institute of Botany, Jiangsu Province and Chinese Academy of Sciences (Nanjing Botanical Garden Memorial Sun Yat-Sen), Nanjing 210014, China; d Department of Agriculture, Ministry of Local Government and Rural Development, AJ 2, Ajumako, Central Region, Ghana; e Innovation Academy for Precision Measurement Science and Technology, Chinese Academy of Sciences, Wuhan 430071, China

**Keywords:** Antibiotic resistance genes, Nonylphenol, Conjugative transfer, Public health

## Abstract

Given that many organic pollutants have been reported to facilitate the plasmid-mediated conjugative transfer of antibiotic resistance genes (ARGs), it was naturally deduced that nonylphenol (NP) can also have this kind of effect. Whereas, this study demonstrates an entirely different result that environmentally relevant concentrations of NP attenuate plasmid-mediated ARGs conjugative transfer (maximum inhibition rate 64 %), further study show that NP exposure had no significant effect on bacterial growth, cell vitality, oxidative stress response, and expression of conjugation-relevant genes, which were reported to closely relate to the conjugative transfer in numerous studies. Conclusively, it was found that the dispersant function of NP impeded the occurrence of cell mating, thus was responsible for the decline of conjugative transfer. This study shows a new perspective on understanding the effect of organic pollutants like NP on the ARGs horizontal dissemination in environment.

## Highlights

• Nonylphenol (NP) attenuate the plasmid-mediated conjugative transfer of ARGs.
• The effect of NP on ARGs conjugation is different from other reported organic pollutants.
• Conventional mechanism cannot demonstrate this phenomenon.
• Dispersant function of NP is responsible for the decline of conjugative transfer.

## Graphical abstract

**Figure:**
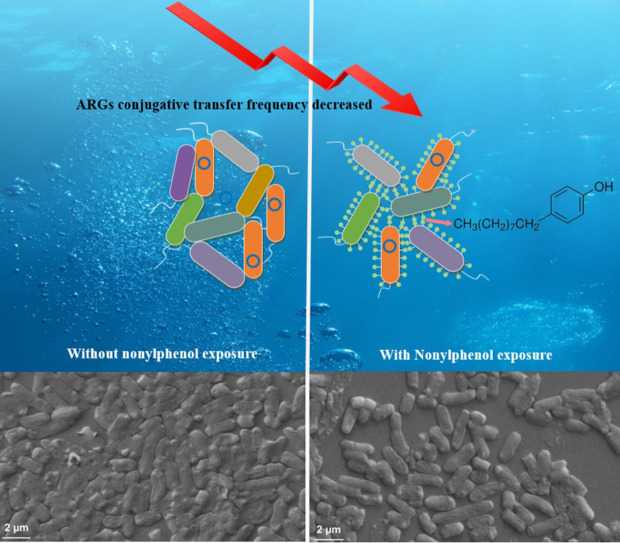

## Introduction

Antibiotic resistance genes (ARGs) in the environment are a global concern, that pose significant threats to human health and ecological balance (Martinez, 2008; Zhuang et al., 2021). Induced by widespread antibiotics use in healthcare, aquaculture, and livestock farms, large quantities of antibiotic resistant bacteria (ARB) and their resistance genes are released into the environments (Pruden et al., 2013). Water bodies, including oceans, rivers, lakes, ponds and groundwater, are plagued by ARGs contamination (Hatos and Martiny, 2015; Yang et al., 2017; Wang et al., 2020; Leff et al., 2023), which was mainly due to the discharge of untreated or inadequately treated wastewater containing antibiotics and ARB (Rizzo, et al., 2013). In addition, runoffs from agricultural fields treated with livestock manure-derived soil amendments can also introduce ARGs into waterbodies (Zhang et al., 2021). Evidently, ARGs persistence in aquatic environments poses a health risk to human beings through water consumption and recreational use (Saibu et al., 2024).

Nonylphenol (NP) serves as nonionic surfactants in various industrial, agricultural, and domestic applications, functioning as surfactants, wetting agents, stabilizers, or emulsifiers. NP finds widespread use in food packaging and daily products, including hair dyes, textile printing, and pesticides, etc. (Soares et al., 2008; Zhang et al., 2023). It's not easily biodegradable in nature, meaning it can persist in the environment over an extended period. NP contamination in water has drawn worldwide concern because it can enter the human body through drinking and polluted aquatic foods consumption (Hong et al., 2020; Bhandari et al., 2021). It can interfere with the endocrine and reproductive systems, to cause infertility, precocious puberty, and abnormal development of sexual organs, especially for children (Soares et al., 2008; Zhang et al., 2023; Cambien et al., 2023). NP is now detected in all kinds of waterbodies and wastewater streams, the concentrations of which range from several nanograms per liter to several hundred nanograms per liter (Ieda et al., 2005; Kim et al., 2005), even more micrograms per liter (Zhang et al., 2012; Lei et al., 2021). Therefore, the combined contamination of ARGs and NP is common in waterbodies. Previous studies showed that many environmentally relevant concentrations of non-antibiotic organic pollutants, such as, sertraline, duloxetine, fluoxetine, bupropion, trichloromethane, dichloroacetonitrile, and prochloraz promoted the plasmid-mediated ARGs conjugative transfer (Ding et al., 2022; Guo et al., 2022; He et al., 2022). So, at first it was hypothesized that environmentally relevant concentrations of NP also had this kind of effect. Whereas, this study demonstrated an entirely different result that environmentally relevant concentrations of NP attenuate plasmid-mediated ARGs conjugative transfer. Whereupon, the mechanisms underlying this phenomenon were explored from cell morphology, physiology, and molecular biology perspectives. This study highlights the different role and mechanisms of NP in influencing the plasmid-mediated ARGs conjugative transfer by comparison with other reported organic pollutants.

## Results and discussion

### Effects of NP exposure on the conjugative transfer

**Fig. 1:**
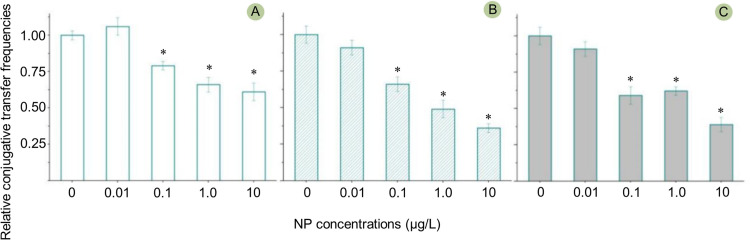
The effects of NP exposure on the ARGs conjugative transfer mediated by the mobile plasmid RP4 at 6 h (A), 8 h (B), and 12 h (C). The error bars represent ± SD (*n* = 5). A statistically significant difference between the control and treatment group (*P* < 0.05) was shown with * Differences between groups were analyzed using the ANOVA tests with post hoc adjustments. SD, standard deviation.

Previous studies documented that many organic pollutants can promote plasmid-mediated conjugative transfer (Ding et al., 2022; Guo et al., 2022; He et al., 2022). Naturally, it was easily deduced that NP had a similar effect, but the result in this study was entirely different. As shown in Fig. 1 and S1, NP exposure significantly decreased the conjugative transfer frequency mediated by ARGs-carrying RP4 plasmid, moreover, the higher the concentration of NP, the more obvious the inhibition. The inhibition effect was more obvious at 8 h and 12 h by comparison with that at 4 h. The lowest conjugative transfer occurred at 10 μg/L of NP, with 0.36 times than that of control.

### Effects of NP exposure on the bacterial growth, cell vitality, and membrane structure

**Fig. 2:**
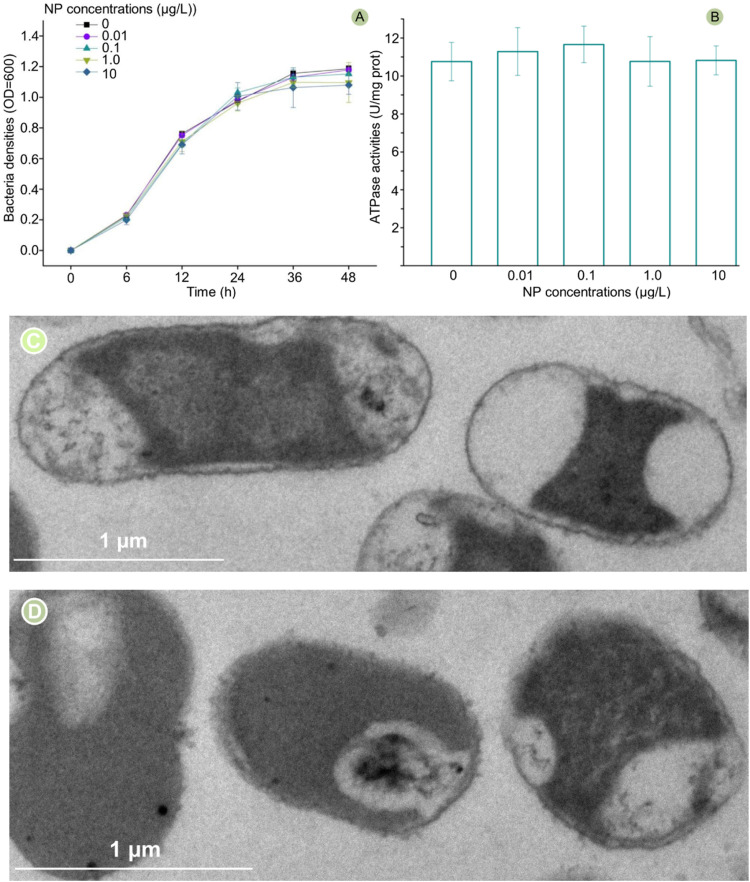
Effects of NP exposure on bacterial growth (A), cell vitality (B), and membrane structure (C, without NP exposure; D, 10 μg/L of NP exposure). The error bars represent ± SD (*n* = 5). A statistically significant difference between the control and treatment group (*P* < 0.05) was shown with * if needed. Differences between groups were analyzed using the ANOVA tests with post hoc adjustments. SD, standard deviation.

In previous studies, the organic pollutants, heavy metals, and nanoparticles intensively influenced the conjugative transfer through interference with bacterial growth, cell vitality, and membrane structure (Qiu et al., 2012; Wang et al., 2020; Ding et al., 2022; Guo et al., 2022; He et al., 2022). This study showed that NP exposure did not significantly affect bacterial growth and cell vitality (ATPase) (Fig. 2A and B), but caused moderate damage to the membrane structure. TEM showed that the membrane contour became relatively fuzzy when exposed to NP compared to the control (Fig. 2C and D). As an important surfactant, the hydrocarbon chain of NP can bond to the hydrophobic group of phospholipid bilayers, interfering with the stricture of the cell membrane, which is a barrier to the plasmid-mediated ARGs horizontal transfer (Qiu et al., 2012; Ding et al., 2022; Li et al., 2022). In other words, moderate damage to the cell membrane contributes to a function-weakened barrier, thus facilitating the ARGs-carrying plasmid transfer from the donor to the recipient (Qiu et al., 2012; Wang et al., 2020; Pu et al., 2021). However, in this study, a function-weakened membrane did not promote conjugative transfer. Thus, there must be other mechanisms which exert a crucial role in attenuating the conjugative transfer.

### Effects of NP exposure on the cellular oxidant and antioxidant properties

**Fig. 3:**
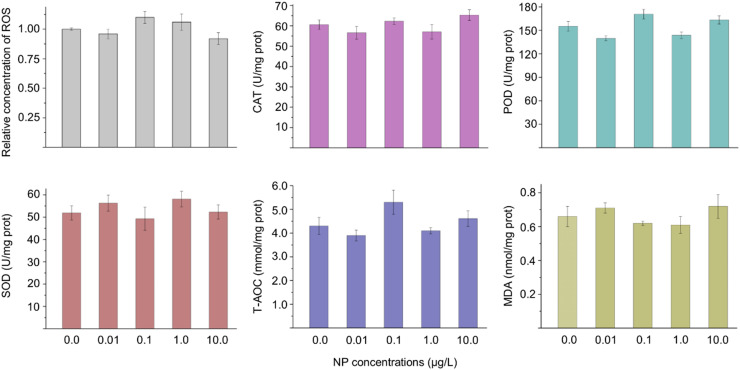
Effects of the different concentrations of NP exposure on the ROS, antioxidant enzymes (SOD, POD, and CAT), T-AOC, and MDA. The error bars represent ± SD (*n* = 5). A statistically significant difference between the control and treatment group (*P* < 0.05) is shown with * if needed. Differences between groups were analyzed using the ANOVA tests with post hoc adjustments. SD, standard deviation. CAT, catalase; POD, peroxidase; SOD, superoxide dismutase; T-AOC, total antioxidant capacity; MDA, malondialdehyde.

When microbes are exposed to environmental stress such as organic pollutants, heavy metals, and nanoparticles, the overaccumulation of ROS can cause peroxidation damage to the cell membrane, which can also weaken the barrier function of a membrane (Qiu et al., 2012; Ding et al., 2022; Li et al., 2022). In this study, the NP exposure had no significant effects on ROS concentration, antioxidase activities (CAT, POD, SOD, and T-AOC), and MDA concentration (Fig. 3). These results demonstrated that the moderate damage to the cell membrane had no relationship with the cellular oxidation system.

### Effects of NP exposure on the expression of conjugation-relevant genes

**Fig. 4:**
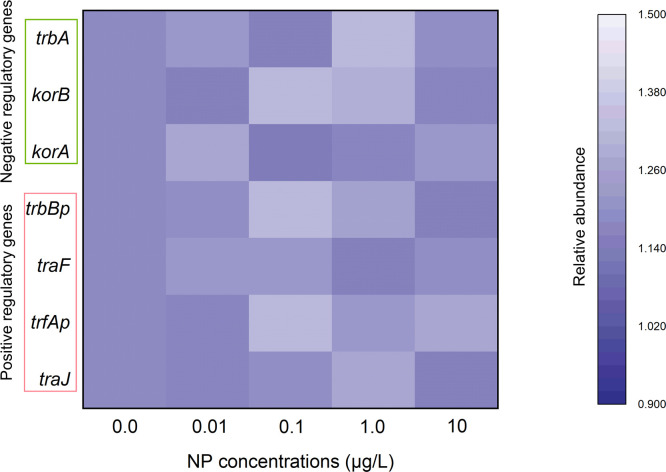
Relative abundance of negative regulatory genes (*korA, korB*, and *trbA*) and positive regulatory genes (*trbBp, traF, trfAp* and *traJ*) to different NP exposure concentrations. A statistically significant difference between the control and treatment group (*P* < 0.05) was shown with * if needed. Differences between groups were analyzed using the ANOVA tests with post hoc adjustments. Mpf, mating pair formation system; Dtr, DNA transfer and replication.

The global regulatory genes *korA, korB*, and *trbA* exert an important role in negatively regulating the RP4-mediated ARGs conjugative transfer (Samuels et al., 2000; Zhang et al., 2018; Li et al., 2022). The expression of *korA* and *korB* can synergistically inhibit the expression of *trfAp*, a promoter which initiates the expression of mating-relevant genes. Another gene *trbA* is also known as playing a negative role in the process of conjugative transfer. The *trbBp* and *traF* are the positive regulatory genes involved in the stage of bacteria mating, while other two genes, *trfAp* and *traJ* play a positive role in the stage of plasmid transfer and replication. Numerous studies have demonstrated that organic pollutants, heavy metals, and nanoparticles, can promote the conjugative transfer through down-expressing negative regulatory genes and up-expressing positive regulatory genes (Qiu et al., 2012; Wang et al., 2020; Ding et al., 2022; Guo et al., 2022; He et al., 2022). However, in this study, the expression of these conjugation-relevant genes performed no obvious changes when exposed to NP (Fig. 4).

### Dispersant function of NP was responsible for the decline of conjugative transfer

Previous studies documented that conjugative transfer increased when exposed to organic pollutants, such as trichloromethane, dichloroacetonitrile, prochloraz, triclosan, sertraline, duloxetine, fluoxetine, bupropion, escitalopram, etc. (Ding et al., 2022; Guo et al., 2022; He et al., 2022). Logically, it was easily deduced that NP can also have this kind of effect. Interestingly, this study showed an entirely different result with the finding that environmentally relevant concentrations of NP attenuated the ARGs conjugative transfer. The possible underlying mechanisms such as bacterial growth, cell vitality, cell membrane, oxidative stress response system, and expression of conjugation-relevant genes which were commonly discussed in the previous studies were investigated in this study. However, the results showed that NP exposure had no significant effects on these aforementioned biological properties, except for cell membrane structure. These results cannot elucidate the phenomenon that why environmentally relevant concentrations of NP attenuated the conjugative transfer.

**Fig. 5:**
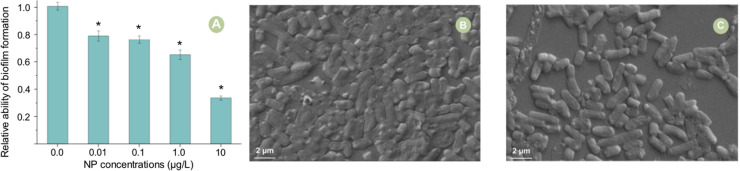
Effects of different concentrations of NP on biofilm formation (A). The mating status of bacteria without (B) and with NP exposure (10 μg/L, C). The error bars represent ± SD (*n* = 5). A statistically significant difference between the control group and treatment group (*P* < 0.05) was shown with *. Differences between groups were analyzed using the using the ANOVA tests with post hoc adjustments.

**Fig. 6:**
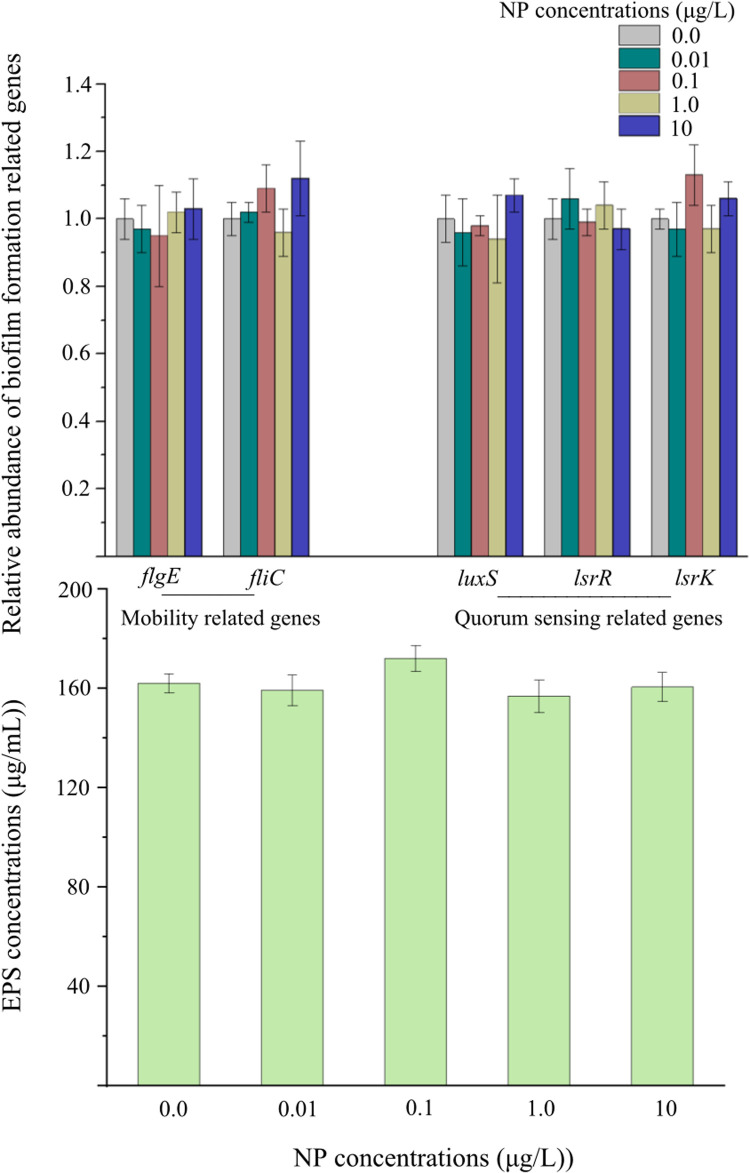
Relative expression abundance of biofilm formation related genes and concentrations of EPS to different NP exposure concentrations. The error bars represent ± SD (*n* = 5). A statistically significant difference between the control and treatment group (*P* < 0.05) was shown with * if needed. Differences between groups were analyzed using the ANOVA tests with post hoc adjustments.

The premise of the occurrence of plasmid-mediated ARGs conjugative transfer is the cell-to-cell contact, so the ability of cell mating is crucial for the occurrence of conjugative transfer (Qiu et al., 2012; Li et al., 2022). Whereupon, the ability of cell mating characterized by the ability of biofilm formation was determined in this study. The result showed that the ability of biofilm formation significantly decreased when exposed to NP, and the higher the concentration of NP was, the more difficult the biofilm formed (Fig. 5A). To validate this result, the SEM experiment was conducted, the images of which showed that fewer cells were in mating status when exposed to NP in comparison to those in the control group (Fig. 5B and C). The cell mobility, extracellular polymers (EPS), and quorum sensing (QS) could contribute to the biofilm formation (Wang et al., 2020; Ding et al., 2022). Whereupon, the effects of NP exposure the expression of two typical flagella-related genes (*flgE* and *fliC*) and three QS-related genes (*luxS, lsrR*, and *lsrK*), as well as the concentrations of EPS were investigated in this study. As shown in Fig. 6, NP exposure exerted no significant effects on these genes expression and the EPS concentration. But what caused the decline in biofilm formation ability? It is common knowledge that NP is a famous dispersant, therefore the dispersibility of bacteria in the conjugation solution was greatly increased when exposed to NP, which impeded the biofilm formation. This change decreased the number of mating cells, thus attenuating the plasmid-mediated ARGs conjugative transfer.

## Conclusions

This study demonstrated that NP exposure attenuated plasmid-mediated ARGs conjugative transfer. While the underlying mechanisms was further explored, the results showed that NP exposure did not change the bacterial growth, cell vitality, oxidative stress response systems and expression of conjugation-relevant genes, which were documented to be closely related to the conjugative transfer in numerous studies. Given that NP has the function of dispersant, the decline in the number of cell-mating between the donors and recipients was responsible for the attenuation of conjugative transfer.

## Materials and methods

### Bacterial strain and culture condition

The study used *Escherichia coli* HB101, carrying a broad host-range conjugative plasmid RP4 as the donor bacterial strain, RP4 plasmid contained resistance genes against ampicillin (Amp), kanamycin (Kan), and tetracycline (Tet). *E. coli* K12 MG1655 with rifampicin (Rif) resistance was used as the recipient bacteria. Lysogeny broth (LB) medium with 30 mg/L Tet for the donors and 30 mg/L Rif for the recipients were used to preculture the bacteria at 37 °C overnight, respectively. After harvesting via centrifuging at 5000 rpm for 5 min, the cells were washed twice with sterile phosphate-buffered saline (PBS, 0.01 mol/L, pH 7.2). The collected cells were then resuspended in PBS, and OD600 was adjusted to 1.0 for ready-to-use.

### Conjugation system

The conjugation system was set according to Li et al. Both of the final concentration of donor and recipient in the conjugation solution were 1 × 10^7^ CFU/mL. The final volume of conjugation system was 30 mL. Environmentally relevant concentrations of NP (4-*n*-NP) were set as 0, 0.01, 0.1, 1.0, and 10.0 μg/L respectively, according to the previous studies. Five parallel conjugation experiments for each group were conducted simultaneously. LB agar plates containing 30 mg/L Rif were used to determine the number of recipients; LB agar plates containing 50 mg/L Amp, 30 mg/L Kan, 30 mg/L Tet, and 30 mg/L Rif were used to determine the number of transconjugants. The conjugative transfer frequency at 6 h, 8 h, and 12 h was presented as the numbers of transconjugants per recipient cells (Ding et al., 2022).

### Determination of the bacterial growth and cell vitality

LB medium was used to culture the donor *E. coli* HB101 and recipient *E. coli* K12 MG1655 at 30 °C. Gradient concentrations of NP were set as 0, 0.01, 0.1, 1.0, and 10.0 μg/L respectively. Five parallel conjugation experiments for each group were conducted simultaneously. The bacterial growth was determined by monitoring the cell density at OD 600 with a spectrophotometer after eight hours conjugation. The activity of ATPase was used to indicate the influence of NP on cell vitality and was determined by a commercial kit (Nanjing Jiancheng Bioengineering Institute, Nanjing, China) according to the manufacturer's instructions.

### Cell morphology

The mating status was captured by scanning electron microscopy (SEM) (EVO LS10, ZEISS, Germany) according to Li et al. (2022). Images of the cell membrane structure were visualized using transmission electron microscopy (TEM) (HT7700 Exalens, Hitachi, Japan) at 200 kV after eight hours conjugation (Qiu et al., 2012). The detailed information of sample preparation is described at Text S1.

### Determination of oxidative and antioxidative properties

A commercial kit (Nanjing Jiancheng Bioengineering Institute, Nanjing, China) containing a fluorochrome of 2,7-dichlorodihydrofluorescein diacetate was used to determine ROS after eight hours conjugation. The ROS concentration was analyzed via a flow cytometer (CytoFLEX, Beckman Coulter, USA). The concentration of malondialdehyde (MDA), and the activities of superoxide dismutase (SOD), peroxidase (POD), and catalase (CAT), and the total antioxidant capacity (T-AOC) were measured also using commercial kits (Nanjing Jiancheng Bioengineering Institute, Nanjing, China) according to the manufacturer's instructions.

### Expression of conjugation-relevant genes, mobility-relevant genes, and QS-relevant genes

Total RNA extraction and cDNA synthesis were according to Ding et al. (2022) and Li et al. (2022). The expression of conjugation-relevant genes, mobility-relevant genes, and quorum sensing (QS)-relevant genes was evaluated using quantitative reverse transcription polymerase chain reaction (qRT-PCR) after eight hours conjugation. Three global regulatory genes that negatively regulate conjugative transfer (*korA, korB*, and *trbA*), two positive regulatory genes involved in mating pair formation (*trbBp* and *traF*), two positive regulatory genes involved in plasmid transfer and replication (*trfAp* and *traJ*), two typical flagella-related genes (*flgE* and *fliC*), and three QS-related genes (*luxS, lsrR*, and *lsrK*) were quantified in this study. The 16S rRNA gene was used to normalize gene expression. The fold-change in gene expression was calculated using the method of 2^−ΔΔCt^ (Livak and Schmittgen, 2001). The PCR primer sets for each target gene are listed in Table S1.

$$-\Delta \Delta \text{Ct}=-({\left(Ct_{\text{NPT}---}Ct_{\text{NPR}}\right)}_{-}--\left(Ct_{\text{CKT}---}Ct_{\text{CKR}}\right))$$

where Ct _NPT_ is the threshold number of target genes in the NP treatment groups; Ct _NPR_ is the threshold number of reference gene (16S rRNA) in the NP treatment groups; Ct _CKT_ is the threshold number of target genes in the control group; Ct _CKR_ is the threshold number of reference gene (16S rRNA) in the control group.

### Quantitative biofilm assay

Quantitative biofilm assay was modified from the method described by Wakimoto et al. (2004). To obtain the biofilm, the medium was gently sucked out after 8 h conjugation, and the biofilm attached to the flask wall was immediately immobilized with methanol, then dyed with 1 % crystal violet solution at room temperature for 5 min. After removing the crystal violet solution, 33 % (v/v) acetic acid was added. The flasks were incubated at 37 °C for 30 min, then the absorbance was measured at 590 nm by spectrophotometer. The sample without bacteria incubation were set as the negative control.

### EPS extraction

EPS extraction was according to the method described by Wang et al. (2020). In detail, conjugation solutions were centrifuged for 15 min (12,000 rpm, 4 °C), and the cell pellets were washed thrice with 0.9 % NaCl solution. Then the cell pellets were resuspended in 10 mL double-distilled water and 8 mL ethylenediaminetetraacetic acid disodium (2 %) for 4 h at 4 °C. Remaining cells were removed by centrifugation of the above supernatant for 30 min (12,000 rpm, 4 °C). Finally, double- distilled water was used to dialyze the acquired supernatant for 24 h and cellulose acetate membrane (0.45 μm) for filtrating. The supernatant was used as the EPS fraction for chemical analyses. The contents of proteins and carbohydrates were determined by Lowry method and anthrone method, the content of nucleic acids was determined using a UV spectrophotometer. The total content of EPS was measured with the sum of the three components.

### Statistical analysis

SPSS Statistics 29.0 (SPSS, Chicago, USA) was used for all data analysis. Differences between groups were analyzed using the ANOVA tests with post hoc adjustments was used to assess significant differences between groups. A value of *p* < 0.05 was considered significant.

## CRediT authorship contribution statement

**Si-Zhou Liang:** Writing – review & editing, Writing – original draft, Methodology, Investigation, Data curation. **Ya-Jun Chang:** Writing – review & editing, Writing – original draft, Project administration, Methodology, Investigation, Data curation. **Philip Semaha:** Writing – review & editing, Writing – original draft, Methodology, Investigation. **Li-Zhu Liu:** Investigation. **Yan Gao:** Writing – review & editing. **Zhi Wang:** Writing – review & editing, Writing – original draft, Validation, Data curation, Conceptualization. **Wei-Guo Zhang:** Writing – review & editing, Writing – original draft, Visualization, Validation, Supervision, Software, Resources, Project administration, Methodology, Investigation, Funding acquisition, Formal analysis, Data curation, Conceptualization.

## Declaration of competing interest

The authors declare that they have no known competing financial interests or personal relationships that could have appeared to influence the work reported in this paper.

## Data Availability

Data will be made available on request

## Appendix Supplementary materials

Image, application 1
