# Supplementary material for: A maverick: Environmentally relevant concentrations of nonylphenol attenuate the plasmid-mediated conjugative transfer of antibiotic resistance genes
Source: Water Res X. 2024 Jul 26;24:100241. doi: 10.1016/j.wroa.2024.100241 (PMC11345678; doi:10.1016/j.wroa.2024.100241)
Supplement: Supplementary file 1 [file mmc1.docx]

**A maverick: environmentally relevant concentrations of nonylphenol attenuate the** **plasmid-mediated conjugative transfer of antibiotic resistance genes**

Si-Zhou Liang ^a,b,1^, Ya-Jun Chang ^c,1^, Philip Semaha ^d^, Li-Zhu Liu ^a^, Yan Gao ^a^, Zhi Wang ^e,^*, Wei-Guo Zhang ^a,b,^*

^a^ *China Ministry of Agriculture Key Laboratory at Yangtze River Plain for Agricultural Environment, Institute of Agricultural Resources and Environment, Jiangsu Academy of Agricultural Sciences, Nanjing 210014, China*

^b^ *School of the Environment and Safety Engineering, Jiangsu University, Zhenjiang 212013, China*

^c^ *Jiangsu Key Laboratory for the Research and Utilization of Plant Resources, Institute of Botany, Jiangsu Province and Chinese Academy of Sciences (Nanjing Botanical Garden Memorial Sun Yat-Sen), Nanjing 210014*

^d^ *Department of Agriculture, Ministry of Local Government and Rural Development, AJ 2, Ajumako, Central Region, Ghana*

^e^ *Innovation Academy for Precision Measurement Science and Technology, Chinese Academy of Sciences, Wuhan, 430071*

^1^ These authors contributed equally to this work

*** Corresponding author**

E-mail: weiguozhang@jaas.ac.cn (W-G. Zhang); [zwang@apm.ac.cn](mailto:zwang@apm.ac.cn) (Z., Wang)

**Text S1.** **Samples Preparation for the SEM and TEM Observation**

Briefly, after 8 hours conjugation, 1 mL of conjugation solution was centrifugated at 5000 rpm g for 5 min. Cell pellet was washed twice with PBS, fixed with 2.5 % glutaraldehyde (v/v) overnight at 4 °C, washed again twice with PBS, and fixed with 1 % OsO4 (v/v) for 4 h. Subsequently, cells were dehydrated with 30 %, 50 %, 70 %, 85 %, 95 %, and 100 % ethanol. The samples used for SEM observation needed replacement with isoamyl acetate, critical point drying, and ion sputtering. The mating status was captured by EVO LS10 (ZEISS, Germany). The samples used for TEM observation were embedded in epoxy and cut into slices. Images of the cell membrane structure were visualized using HT7700 Exalens (Hitachi, Japan) at 200 kV.


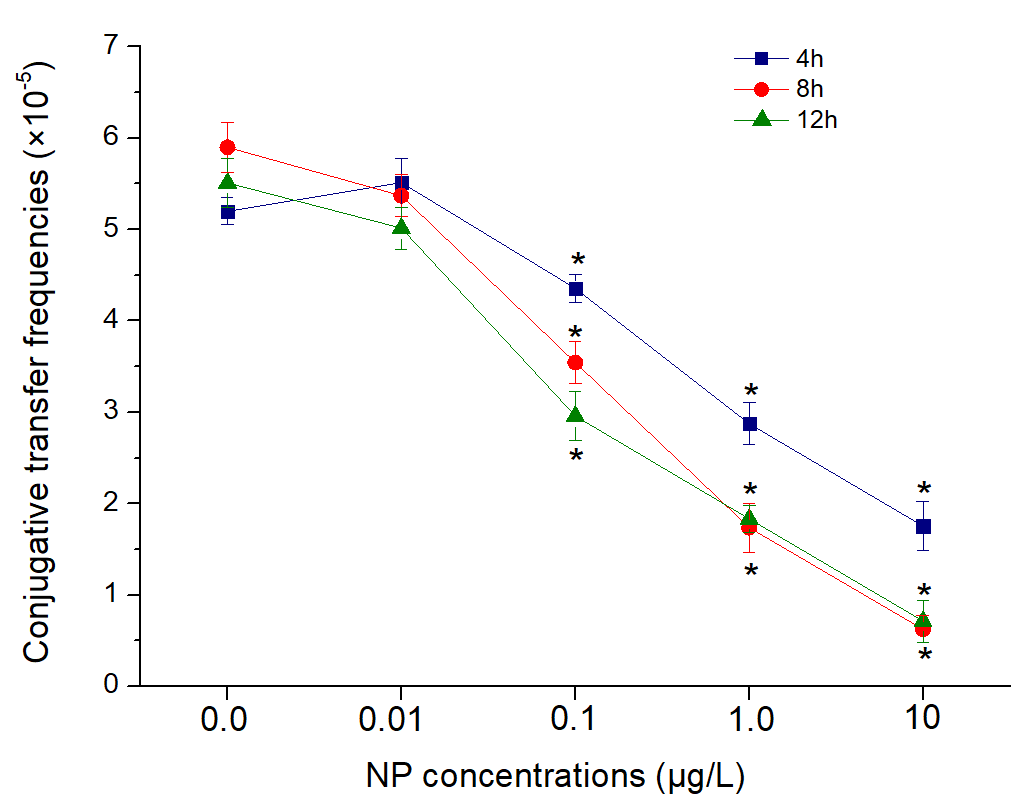


**Figure S1.** The effects of NP exposure on the ARGs conjugative transfer mediated by the mobile plasmid RP4 at 6 h (A), 8 h (B), and 12 h (C). The error bars represent ± SD (*n* = 5). A statistically significant difference between the control and treatment group (*P* < 0.05) was shown with *. Differences between groups were analyzed using the ANOVA tests with post hoc adjustments. SD, standard deviation.

**Table S1.** **Primer sequences used in this study**

| Category | Gene | Primer | Sequence of Primer (5’-3’) |
| --- | --- | --- | --- |
| Global regulatory genes | *korA* | *korA-F* | TCGGGCAAGTTCTTGTCC |
|  |  | *korA-R* | GCAGCAGACCATCGAGATA |
|  | *korB* | *korB-F* | CTGGTCGGCTTCGTTGTA |
|  |  | *korB-R* | TGAAGTCACCCATTTCGGT |
|  | *trbA* | *trbA-F* | TGGAAACTCCCCTACCTCTT |
|  |  | *trbA-R* | CCACACTGATGCGTTCGTAT |
| Mating pair formation system genes | *trbBp* | *trbBp-F* | CGCGGTCGCCATCTTCACG |
|  |  | *trbBp-R* | TGCCCGAGCCAGTACCGCCAATG |
|  | *traF* | *traF-F* | GGCAACCTCGTCGCCTTTA |
|  |  | *traF-R* | GCAAGTCGGCGTGTTTTCG |
| DNA transfer and replication system genes | *trfAp* | *trfAp-F* | GAAGCCCATCGCCGTCGCCTGTAG |
|  |  | *trfAp-R* | GCCGACGATGACGAACTGGTGTGG |
|  | *traJ* | *traJ-F* | GCCCGTGATTTTGTAGCCC |
|  |  | *traJ-R* | TGAAACCAAGCCAACCAGGAA |
| Flagella related genes | *flgE* | *FlgE-F* | CCTGGTGAATATGCAAGGTTTA |
|  |  | *FlgE-R* | ATTGACGCCGTGGTGGTAGTT |
|  | *fliC* | *fliC-F* | ATTAACAGCGCGAAGGATGACG |
|  |  | *fliC-R* | TACCGTCAGTTCACGCACACG- |
| Quorum sensing related genes | *luxS* | *luxS-F* | ATGAGCAGCGTGTTGCTGATGC |
|  |  | *LuxS-R* | CAACGAGTGCATCTGGTAAGTGC |
|  | *lsrR* | *LsrR-F* | TATCAGCCAGGGCGAACAGT |
|  |  | *LsrR-R* | ATGGTCTTCAGCGCGCTTAA |
|  | *lsrK* | *LsrK-F* | TGGCGGTGGATCCCTCTAA |
|  |  | *LsrK-R* | AACTTACCACGCCCAGCAAT |
| 16S rRNA | *16S rRNA* | *16s-F* | CCTACGGGAGGCAGCAG |
|  |  | *16s-R* | ATTACCGCGGCTGCTGG |
